# Oxidative Stress and Natural Products in Orthodontic Treatment: A Systematic Review

**DOI:** 10.3390/nu16010113

**Published:** 2023-12-28

**Authors:** Francesco Inchingolo, Angelo Michele Inchingolo, Giulia Latini, Laura Ferrante, Irma Trilli, Gaetano Del Vecchio, Giulia Palmieri, Giuseppina Malcangi, Alessio Danilo Inchingolo, Gianna Dipalma

**Affiliations:** Interdisciplinary Department of Medicine, University of Bari “Aldo Moro”, 70124 Bari, Italy; francesco.inchingolo@uniba.it (F.I.); angeloinchingolo@gmail.com (A.M.I.); dr.giulialatini@gmail.com (G.L.); lauraferrante79@virgilio.it (L.F.); trilliirma@gmail.com (I.T.); dr.gdelvecchio@gmail.com (G.D.V.); giuliapalmieri13@gmail.com (G.P.); ad.inchingolo@libero.it (A.D.I.); giannadipalma@tiscali.it (G.D.)

**Keywords:** natural products (N.P.s), oxidative stress (O.S.), orthodontic

## Abstract

In recent years, orthodontics, a specialized branch of dentistry, has evolved considerably in terms of both techniques and materials used. Aimed at correcting dental malocclusions and craniofacial anomalies, it improves the functionality and aesthetics of the face and oral cavity. However, orthodontic treatment, in its developmental stages, may induce oxidative stress (O.S.) phenomena, with an increase in the production of reactive oxygen species (ROS), damaging the dental and periodontal tissues involved, affecting the short-, medium- and long-term results. Studies on the antioxidant effects of natural products (e.g., resveratrol, green tea, turmeric, etc.) in the medical field have aroused considerable interest in recent years. A systematic literature review was conducted on the PubMed, Scopus, and Web of Science databases using natural products (N.P.s), O.S., and orthodontic as keywords. The study aims to consider the determinants of the increase in ROS occurring during orthodontic treatment and the possibility of natural products being able to control and neutralize biochemical phenomena by restoring the physiological process in which the balance between the production of ROS and the ability of the body’s antioxidant system to neutralize them is in favor of the latter.

## 1. Introduction

**Figure 1:**
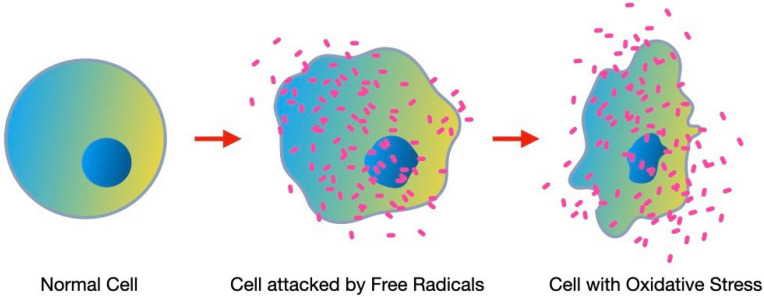
Cellular aging following O.S.

Orthodontics represents an essential branch of modern dentistry, aimed at correcting dental malocclusions and craniofacial anomalies in order to improve the functionality and aesthetics of the oral cavity [1]. Over the years, orthodontics has made significant progress both in terms of techniques and materials used [2]. However, despite considerable progress, orthodontic treatment can cause physical and metabolic stress for the dental and periodontal tissues involved, leading to oxidative stress (O.S.) phenomena [3]. O.S. is a physiological process in which the equilibrium between reactive oxygen species generation (ROS) and the body’s antioxidant system’s capacity to counteract them is weakened in favor of ROS [4,5]. Biomolecules found in cells, such as proteins, lipids, and nucleic acids, can sustain harm from these unstable compounds, leading to pathological conditions and accelerated cellular aging [6,7] (Figure 1).

O.S. can be caused by a variety of factors, including the following:

- Pathologies: Some diseases, such as rheumatoid arthritis, diabetes, cardiovascular diseases, and some neurodegenerative diseases, are associated with an increase in O.S. This may be due to an alteration in the equilibrium between the production of ROS and the body’s ability to neutralize them using natural antioxidants [8,9,10].
- Lifestyle: Exposure to environmental stressors, such as tobacco smoke, air pollution, alcohol, and poor diet, can contribute to increased O.S. The excessive intake of antioxidants can also alter the body’s redox balance [11,12].
- Infections and inflammation: Chronic infections and inflammatory processes can increase the production of ROS as the immune system attempts to fight the infection or respond to inflammation [13,14].
- Medical treatments: Some medical treatments, such as radiation therapy and chemotherapy, can temporarily increase O.S. in the body’s cells in an attempt to destroy cancer cells [4,9,15,16].

**Figure 2:**
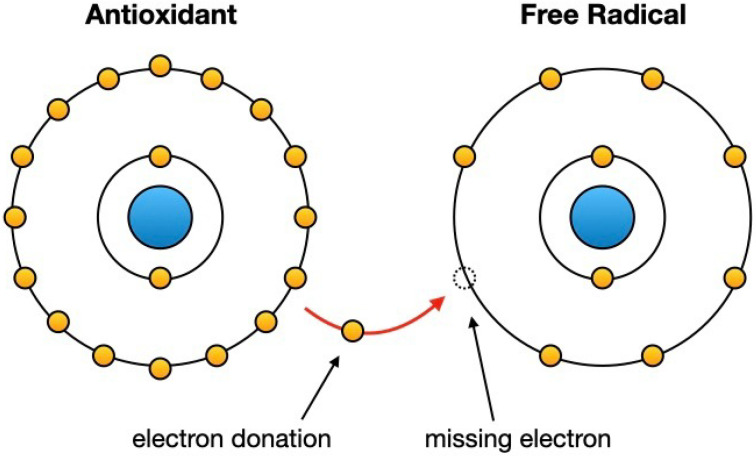
The action of antioxidants makes free radicals no longer reactive, transforming them into more stable molecules.

The O.S. process can be triggered by various factors, including mechanical pressure on the teeth, inflammation associated with tooth movement, and the use of fixed orthodontic appliances [17,18]. The use of orthodontic appliances, such as braces, often involves the presence of brackets, wires, metal clasps, etc. [19]. These materials can react with saliva and oral fluids, generating metal ions that promote the generation of free radicals [20] (Figure 2).

The movement of teeth through the application of mechanical forces creates friction between the teeth themselves and between the teeth and the components of the orthodontic appliance [21]. This friction can cause O.S. in surrounding tissues [22,23]. Orthodontics can cause gum irritation and inflammation due to the buildup of bacterial plaque around orthodontic appliances. It is also caused by difficulty in cleaning due to the presence of the devices themselves [3]. This inflammation may contribute to increased O.S. in the oral cavity [3,24]. Mechanical pressure induces a series of biological involvements of the local inflammatory response and the remodeling of the surrounding bone tissue [25,26]. Initially, compression of the bone and periodontal cells causes the release of inflammatory mediators such as tumor necrosis factor-alpha (TNF-α) and interleukin-1 beta (IL-1β) [4]. These mediators can trigger a chain reaction leading to increased ROS production. ROS, such as hydrogen peroxide (H_2_O_2_), superoxide (O_2_^−^), and nitric oxide (NO), can overcome the capacity of cellular antioxidant defenses and cause damage to proteins, DNA, and lipids [27]. Studies suggest that osteoblastic and osteoclastic cells involved in the bone remodeling process are particularly sensitive to oxidative damage [3,28].

Excessive ROS production can inhibit osteoblast differentiation and increase osteoclast activity, thus compromising the balance between bone formation and resorption [29,30]. This imbalance can lead to delays in tooth movement and post-treatment instability [29]. Moreover, in terms of the mechanical aspect, fixed orthodontic appliances can constitute a favorable environment for the proliferation of bacteria and dental plaque [29,31]. When plaque is present, more acids are produced by bacteria, creating an acidic environment that damages tooth enamel and can contribute to increased O.S. Furthermore, orthodontic device components that irritate the oral mucosa may produce a reaction [32]. Accumulating evidence suggests that O.S. associated with orthodontics may have significant clinical implications [33,34]. Additionally, chronic inflammation, caused by orthodontic appliance irritation, can lead to gingivitis and periodontitis, with potentially serious consequences for long-term oral health [17,35,36,37,38]. In the context of clinical challenges related to O.S. in orthodontics, therapeutic approaches based on natural products have attracted new interest [39,40]. Natural products, often derived from plants, are known for their antioxidant and anti-inflammatory properties [41]. These drugs have the property to be very effective in reducing O.S. associated with orthodontics, promoting oral healing and well-being. The natural products with antioxidant properties are coenzyme Q10 (CoQ10), vitamin E, vitamin C, and selenium. CoQ10 participates in the electron transport pathway of the mitochondria and is known for its ability to reduce ROS levels preserving the viability of osteoblastic cells exposed to mechanical forces similar to those generated during orthodontic treatment [42]. Vitamin E is a fat-soluble antioxidant that can protect cellular lipids from oxidative damage. Water-soluble vitamin C is an antioxidant that may counteract reactive oxygen species (ROS) and is crucial to produce collagen, essential for periodontal health. Selenium is a key component of antioxidant proteins, including glutathione peroxidase, which contributes to the defense against ROS [43]. Plant extracts, such as aloe vera, chamomile, and myrrh, have been studied for their anti-inflammatory and antioxidant properties. These extracts can be used in topical formulations to reduce oral inflammation and O.S. [44,45]. Furthermore, the antibacterial and anti-inflammatory properties of essential oils, including peppermint, lavender, and tea tree oils, are well-known and they may be helpful in preventing infections and relieving inflammation associated with braces [17,18]. Antioxidants are chemicals that assist in scavenging free radicals. Some natural products, such as green tea, are known for their antioxidant properties and can be used in toothpaste or oral supplements. An adequate intake of calcium and vitamin D is essential for dental health. Calcium can help strengthen tooth enamel, while vitamin D is important for calcium absorption. These nutrients can be obtained from natural sources such as dairy products and sun exposure. Important bioactive substances present in propolis include ascorbic acid, organic acids, flavonoids, carotenoids, phenolic acids, enzymes, and other proteins [46]. The field of dentistry is beginning to appreciate the potential benefits of bee products as antioxidants [47,48]. Honey is made up of nutrients such carbohydrates, proteins, phenols, amino acids, vitamins, minerals, iron, and copper. They have antibacterial, prebiotic, antimutagenic, and antioxidant properties [49]. Due to their antioxidant and anti-inflammatory qualities, phenols may be able to protect oral tissues from oxidative damage, promoting healing and lowering orthodontic treatment-related problems such mouth ulcers [50,51,52]. Fruits, vegetables, tea, wine, and honey are just a few foods that contain polyphenols, which are chemical molecules that have been shown to have anti-inflammatory and antioxidant qualities. This might be useful in reducing orthodontic-related inflammation, particularly when fixed appliances are being used, which can irritate soft tissues [3]. Unquestionably, phenols, or natural antioxidants, are non-enzymatic antioxidants. Through the redox equilibrium restoration and sequestration of ROS, they function as anti-inflammatories. As a result, blocking the synthesis of chemical mediators (TNF-α, IL-8, IL-6, COX, and LOX) lessens inflammatory reactions in cells and the harm they cause. Additionally, polyphenols possess antioxidant properties, which may help shield oral tissues from oxidative damage, promoting healing, and lowering orthodontic treatment-related problems including dental ulcers [53,54]. Their therapeutic advantages need more study to fully understand their unique application in orthodontics [55]. In conclusion, O.S. in orthodontics can be a significant concern to pay attention to. Antioxidants are taken orally in the form of nutraceuticals, nutritional supplements, or vitamin supplements. Natural antioxidants that may be used topically in the form of mouthwashes, gels, pastes, chewing gum, or lozenges are becoming more and more popular [56,57]. These topical antioxidants can aid in lowering ROS, which can function as inflammatory mediators in the emergence of periodontal and gum diseases [58]. The clinical application of these products should be carefully considered, taking into account the concentration, mode of administration, and duration of treatment [59]. Furthermore, it is essential to monitor patients’ reaction and tolerance to natural products, as some people may be sensitive or allergic to certain substances [31,60]. Most natural products used in dentistry have demonstrated a high safety profile, but it is essential to inform patients about the benefits and possible risks associated with the use of such products [61]. It is essential to conduct effective communication with patients, explaining to them the potential benefits of natural antioxidants in their orthodontic therapy [62,63]. O.S. represents a potential complication in orthodontic treatment, with significant clinical implications for patients [15]. Future challenges include conducting large-scale randomized clinical trials to confirm the efficacy and safety of natural products in orthodontics [4,15,64]. Furthermore, dental professionals and patients must be educated on the potential of natural products as a complement to conventional orthodontic treatments [2]. In summary, the management of O.S. in orthodontics represents an emerging challenge, but natural products may offer a promising avenue to address it [17]. The combination of scientific and clinical knowledge can pave the way for more effective and comfortable orthodontic treatment, with the added benefit of preserving long-term oral health [17,65].

The results in the study by Andrea Ballini et al., 2019 show that oral treatment with probiotics can safely and effectively reduce OS in individuals with inflammatory bowel diseases, which include Crohn’s Disease and Ulcerative Colitis [66,67].

## 2. Materials and Methods

### 2.1. Protocol and Registration

This comprehensive analysis was completed following the guidelines in the Preferred Reporting Items for Systematic Reviews and Meta-analysis (PRISMA) 2020 statement [68]. The review protocol was registered at PROSPERO under the unique number 477660.

### 2.2. Search Processing

“Natural Products”, “Oxidative Stress”, and “Orthodontic” were the search terms utilized on the databases (Scopus, Web of Science, and Pubmed) to select the papers under evaluation, with the Boolean operators “AND” and “OR”. The search was limited to just products published in English in the preceding ten years (September 2013–September 2023) (Table 1).

### 2.3. Eligibility Criteria

The reviewers, who worked in pairs, chose works that satisfied the following criteria for inclusion: (1) human subjects-only research; (2) clinical studies or case reports; and (3) research conducted on people taking natural products during orthodontic treatment.

Exclusion criteria were (1) in vitro studies; (2) animal studies; and (3) systematic reviews, narrative reviews, and meta-analyses. Duplicate studies were removed manually.

The review was conducted using the PICO criteria:

- Population: Adults and children, both male and female, who took natural products during orthodontic treatment.
- Intervention: Natural products during orthodontic treatment.
- Comparison: Orthodontics without natural products.
- Outcome: Effectiveness of the natural products in orthodontic treatment, concerning O.S. caused by orthodontic materials or tooth movement.

### 2.4. Data Processing

The screening process allowed for the exclusion of any publications that did not fit the themes examined. It was carried out by reading the article titles and abstracts selected in the earlier identification step. After being found to meet the predefined inclusion criteria, the full text of publications was read. Disagreements among reviewers on the selection of the article were discussed and resolved.

## 3. Results

**Figure 3:**
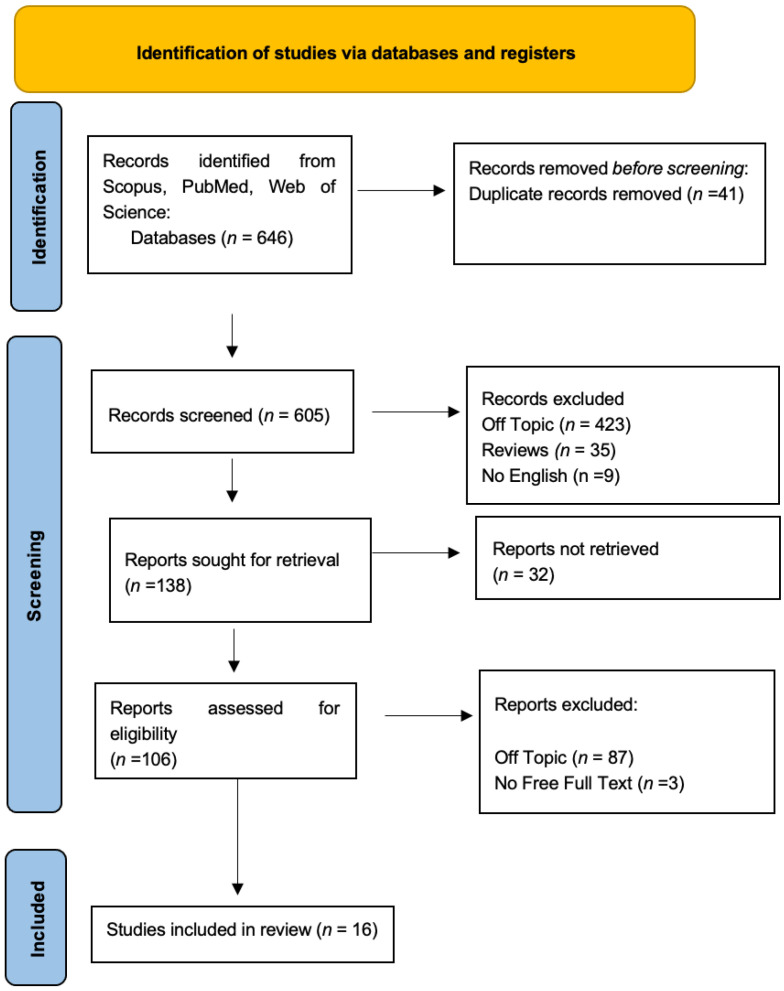
Diagram of the inclusion process using PRISMA flowchart. The Preferred Reporting Items for Systematic Reviews and Meta-Analyses (PRISMA) flow diagram for the literature search.

Keyword searches of the Web of Science (95), Scopus (0), and Pubmed (551) databases yielded a total of 646 articles. After that, 41 duplicates were removed, and 605 articles were included. A total of 574 of these 590 research studies were disqualified for violating the inclusion criteria that had been previously established. Following the screening process, 16 publications were chosen for this work (Figure 3). Each study’s findings are listed in Table 2.

### Quality Assessment and Risk of Bias

**Figure 4:**
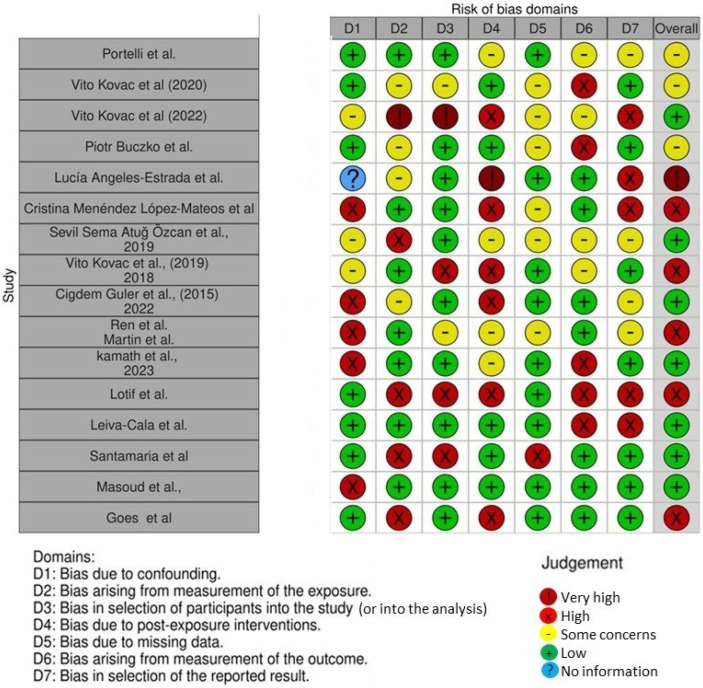
Robins tool to evaluate bias [17,69,70,71,72,73,74,75,76,77,78,79,80,81,82,83].

The risk of bias in the included studies is reported in Figure 4. Regarding the bias due to confounding, most studies have a dubious risk. The bias arising from measurement is a parameter with a low risk of bias. Many studies have a low risk of bias due to bias in the selection of participants. Bias due to post exposure cannot be calculated due to high heterogeneity. The bias due to missing data is low in many studies. The bias arising from the measurement of the outcome is low. Bias in the selection of the reported results is high in most studies. The final results show that four studies have high risk of bias, one has a very high risk of bias, and seven have a low risk of bias.

## 4. Discussion

### 4.1. O.S. and Orthodontic Treatment

O.S. has gained significant attention in both experimental and clinical medicine as a pivotal trigger for various human diseases. It is a pathological condition that exerts severe damage to tissues and cells because of an unbalanced oxidizing agent mixture and antioxidants, leading to alterations in the oxidation–reduction equilibrium. At the core of O.S. lies the free radicals, highly reactive chemical entities in their outer orbitals, where they have single electrons. Free radicals can initiate chain reactions, inflicting substantial harm to cellular proteins and nucleic acids. Among the most notable free radicals are ROS, which play a crucial role in the redox balance [4].

The O.S. can be caused, during orthodontic therapy, by two distinct circumstances present: the device itself and the biomechanics of tooth movement [74]. Orthodontic devices, such as mini-screws and clear aligners, can cause increased O.S. levels. The placement of orthodontic mini-screws involves perforation of the soft tissue and can lead to a local inflammatory response. This inflammation can increase the release of free radicals, contributing to O.S. Clear aligners, on the other side, made of plastic materials, can release chemicals when exposed to certain conditions such as high temperatures. These chemicals could contribute to O.S. if they are absorbed by the oral tissues [84].

The study by Portelli et al. sought to assess O.S. in the saliva of patients receiving self-ligating multibracket vestibular orthodontic treatment. The research aimed to investigate the influence of orthodontic treatment on oral hygiene and its subsequent effect on O.S. Salivary samples were collected at different times during the treatment process [69].

The results indicated that multibracket orthodontic therapy, particularly with self-ligating vestibular appliances, did not significantly influence O.S. within the oral cavity during the first 10 weeks of treatment. In addition, the study revealed improved dental hygiene over the course of orthodontic therapy, likely attributable to patient education and diligent monitoring [85]. These results highlight the potential compatibility of multibracket orthodontic treatment with oral health, dispelling concerns about increased O.S. [69].

Fixed orthodontic appliances, including brackets, archwires, and bands, are essential tools in modern dentistry. However, their long-term safety and biocompatibility remain of utmost importance. Speaking of that, recent research by Kovac in 2020 explains the cytotoxicity and potential for the O.S. of metal ions to be generated by orthodontic equipment: although extremely high concentrations of metal induce cytotoxicity and O.S., this study suggests that the risk to users of orthodontic treatments with fixed appliances is relatively low. However, localized increases in reactive oxygen species may occur inside the mouth, particularly in individuals with deficient antioxidant defense systems, warranting further investigation. Indeed, the use of the model organism *S. cerevisiae* has provided valuable insights into the cytotoxic properties of orthodontic materials and the importance of considering metal composition and biological responses [70].

The same author published another study in 2022 in which he analyzed the release of metal ions from various orthodontic alloys during a 90-day exposure to artificial saliva and evaluated their potential to induce O.S.

The study revealed that metal ion concentrations released from orthodontic alloys remained well below recommended dietary intake levels throughout the 90-day observation period. However, it is important to note that nontoxic concentrations of metals can still have biological effects on oral cells. Hypersensitivity to metals, particularly nickel, should be considered when treating orthodontic patients.

Significant variations in metal ion release have been observed among different orthodontic alloys. Nickel-titanium (Ni-Ti) arches showed significantly higher ion release than stainless steel arches, consistent with previous research. These results underscore that while metal composition plays a role in ion release, alloy quality, surface topography, and manufacturing techniques also have an impact on corrosion resistance [71].

In the 2017 study with 37 healthy nonsmoking participants, researchers Buckzo et al. focused on assessing the impact of fixed orthodontic braces on O.S. and antioxidant response by collecting whole unstimulated and stimulated saliva from participants, both before and after the application of fixed orthodontic braces. The appliances included steel brackets containing nickel, chromium, and other elements [72,86].

Clinical examinations were conducted at three time points: before appliance application, one week after treatment, and twenty-four weeks after application. These examinations included assessments of dental and gingival health. The collected samples were analyzed for various markers of O.S. and antioxidant status.

The study’s findings showed that both unstimulated and stimulated saliva had significantly higher levels of O.S. indicators, such as thiobarbituric acid reactive substances (TBARSs) and total oxidant status (TOS), one week following orthodontic treatment. The concentration of total protein was also elevated twenty-four weeks after appliance application. In terms of antioxidants in unstimulated saliva, no significant changes were observed [72].

In addition, one week after treatment, there was an increase in the concentration of nickel in saliva. The study also showed a positive correlation between nickel concentration and OSI in both unstimulated and stimulated saliva. The findings imply that orthodontic therapy using appliances containing nickel may change the clinically healthy patients’ saliva’s oxidative–antioxidative balance. However, markers of oxidative stress returned to baseline levels twenty-four weeks after treatment, indicating potential mechanisms of adaptation or repair.

These findings underscore the need for further research with larger samples and consideration of other risk factors to better understand the impact of orthodontic interventions on saliva composition [72]. Estrada et al. focused on evaluating the results obtained from the impact of fixed orthodontic appliances on O.S. and genotoxicity in oral epithelial cells.

Sample collection from the 51 volunteer study subjects involved obtaining oral epithelial cells from each subject by rinsing the oral cavity with water and gently scraping the cheeks with a cytology brush. These samples were then stored for nucleic acid extraction. Samples were collected before treatment and at 6 and 9 months after fixed orthodontic treatment. Fixed orthodontic appliances included stainless steel and stainless steel-nickel-titanium wires. Clinical evaluations were conducted throughout orthodontic treatment.

Oxidative DNA damage was assessed by measuring 8-hydroxy-2′deoxyguanosine (8-OHdG) levels before treatment and at 6 and 9 months after orthodontic treatment.

The results showed that O.S. and genotoxic damage in oral epithelial cells changed with the long-term use of fixed orthodontic appliances. The data suggest a biological adaptation response to orthodontic treatment that occurs after approximately 6 months, as indicated by alterations in antioxidant enzyme expression and DNA degradation and instability. This study contributes to the understanding of the effects of orthodontic treatment on oral health and underscores the importance of long-term investigations to comprehensively assess DNA damage induced by fixed orthodontic appliances [73].

In order to analyze the impact of orthodontic treatment on salivary O.S. markers, Cristina Menéndez López-Mateos et al. analyzed patients undergoing different treatment modalities. The study adhered to ethical guidelines and involved 48 patients treated with clear aligners (Invisalign^®^, Align Technology, Santa Clara, CA, USA) and 19 patients treated with 0.22″ Damon System^®^ self-ligating brackets applying light forces. During the 90-day period, we monitored advanced oxidative protein products (AOPPs), total antioxidant capacity (TAC), and myeloperoxidase (MPO) activity in their saliva samples. Surprisingly, we observed that both orthodontic techniques, clear aligners and self-ligating brackets, resulted in increased O.S., as indicated by a significant increase in AOPP levels after the first 30 days of treatment. However, no substantial changes in TAC and MPO levels were noted during the same period, suggesting that the antioxidant capacity of saliva remained relatively stable. Furthermore, it is noteworthy that the differences in AOPP levels between the two treatment groups were not statistically significant during the first 90 days, highlighting the similarity of their impact on O.S. These results indicate that light-force orthodontic treatments may induce O.S. in the early stages of treatment. In addition, this study contributes valuable insights into the biochemical changes that occur in the oral cavity during orthodontic interventions with different techniques [74].

In the study by Sevil Sema Atuğ Özcan et al., various biochemical parameters such as IL-1β, TNF-α, MDA, nitrite, and 8-OHdG were analyzed in orthodontic patients with fixed multibracket therapy. The results of the study revealed that with the exception of IL-1β in GCF at the sixth month of treatment, none of the other biochemical parameters measured in both saliva and GCF showed statistically significant changes at any measurement point [87]. Therefore, it can be concluded that orthodontic tooth movement and orthodontic materials used in treatment did not cause alterations beyond physiological limits suggestive of oxidative damage in either GCF or saliva [75].

Vito Kovac and his collaborators investigated the effects of orthodontic treatment with fixed braces on systemic O.S. parameters in 54 subjects who required orthodontic treatment for mild crowding and misalignment of teeth. Exclusion criteria were applied to ensure homogeneity and eliminate potential confounding factors. Subjects were divided into two groups, the treatment group (TG) and the control group (CG).

Throughout the study, both groups adhered to similar dietary and lifestyle guidelines. The fixed orthodontic appliances used in the TG included stainless steel brackets and nickel-titanium arches. O.S., characterized by the balance between ROS and antioxidative defense (AD), was assessed by capillary blood analysis. The results revealed a short-term systemic increase in ROS levels and the ROS/AD ratio that occurred 24 h after the start of orthodontic treatment in the TG. However, these levels returned to normal within 7 days. Potential factors inducing this O.S. include metal ion exposure, periodontal inflammation, and aseptic inflammation of the periodontal ligament due to the application of mechanical force.

In conclusion, fixed orthodontic treatment can induce systemic O.S., especially in the short term [17].

The study by Cigdem Guler et al. aimed to evaluate the effects of different orthodontic composites on salivary markers in healthy children (11–17 years old) undergoing fixed orthodontic treatment. The study used three orthodontic composites: Transbond XT, Kurasper F, and GrenGloo.

Saliva samples were collected at three time points: before treatment (T1), 1 month after braces placement (T2), and 3 months after braces placement (T3). The levels of total oxidant state (TOS), total antioxidant state (TAS), and 8-hydroxy-2′-deoxyguanosine (8-OHdG) were assessed. The study showed that different orthodontic composites had no significant impact on TOS, TAS, and 8-OHdG levels (*p* > 0.05). TAS levels decreased over time, with significant reductions in the Kurasper F and GrenGloo groups (*p* < 0.05). 8-OHdG levels decreased between T1 and T2 but increased from T2 to T3, with significant differences in the Kurasper F and GrenGloo groups (*p* < 0.05). Overall, the study concluded that the orthodontic composites used in the study had no cytotoxic effects on children, suggesting their safety for use in orthodontic treatment. The observed changes in TAS and 8-OHdG levels can be attributed to factors such as fluoride release and composite content. These results contribute to the understanding of the safety and potential effects of orthodontic composites in pediatric dental care [76].

When mechanical pressures are applied to the teeth during treatment, numerous inflammatory mediators (cytokines) are generated, leading to aseptic inflammation of the periodontal ligament [88]. Consequently, the periodontal ligament undergoes a series of events related to tissue remodeling and tooth movement, causing increased O.S. which is therefore linked to proinflammatory markers as a biological response to orthodontic therapy [89].

Periodontal inflammation is also influenced by the biomechanical movement of the teeth. Orthodontic forces have historically been classified as “light” or “heavy”. At present, self-ligating brackets with superelastic archwires and clear plastic aligners are the most often used light-force, more physiologic orthodontic procedures [74,90].

Light-force orthodontic treatments appear to have no effect on oxidative stress during the first thirty days of therapy. During this first phase of therapy with transparent aligners, modest forces are used to activate and prepare the periodontal ligament for subsequent tooth movement. Low-friction self-ligating brackets showed similar behavior to aligners. After the first thirty days of therapy with transparent aligners and with self-ligating brackets that exert light forces, an increase in. O.S is found [74].

In summary, there are two different ways that orthodontic therapy might cause an increase in O.S.: the device itself and the biomechanics of tooth movement.

The reviewed articles demonstrate how the permanence of orthodontic devices in the mouth causes an increase in O.S. caused by both soft tissue inflammation due to plaque accumulation and soft tissue trauma due to the device itself (e.g., mini-screw positioning).

It has been shown that the metal ions and plastic chemicals release increase the O.S., while remaining well below the recommended dietary intake levels. It is essential to remember that oral cells can still be biologically affected by harmless metal concentrations, especially in patients with hypersensitivity to metals, particularly nickel.

Regarding the biomechanical tooth movement, light forces exerted by self-ligating brackets with superelastic archwires and clear plastic aligners induce less O.S. than the heavy forces of traditional brackets, especially in the early stages of treatment.

Furthermore, there is evidence of a biological adaptation response to orthodontic treatment’s O.S. that occurs after approximately 6 months.

### 4.2. O.S. and Natural Products

Speaking of natural antioxidants is akin to discussing compounds possessing anti-inflammatory and antibacterial properties.

Inducing a natural inflammatory response in the body, either from endogenous adverse stimuli or those originating from the external environment, protects it against infections, hazardous chemicals, and cell damage or death while aiding in the healing and repair process of the afflicted tissue. On the other hand, an unchecked inflammatory response may result in irreparable cellular damage up to necrosis. The oral microbiota is the collection of microorganisms that colonize the oral cavity. Biofilm-forming bacteria in the oral cavity, such as Streptococci and others, can cause numerous infectious diseases such as caries, gingivitis, periodontitis, root canal infections, and alveolar osteitis.

Oral mucosal infections are regulated by different mechanisms than those of biological fluids and can be influenced by the components of saliva, which play an antibacterial and antifungal role [91]. Lactoferrin is a protein found in many biological fluids, including saliva. It is crucial for natural oral immunity as it protects tissues from pathogenic bacteria, fungi, and viruses [74]. The concentration of free and available iron in saliva can influence aggregation and bacterial biofilm formation, playing an important role in oral infections [92]. Oral diseases occur when there is a change in the composition of bacterial plaque and saliva components, compromising the commensal bacterial barrier and promoting the colonization of pathogenic bacteria. Iron accumulation can facilitate infection and the inflammatory process by inhibiting the synthesis of proteins such as ferroportin [93]. Certain types of bacteria and microorganisms are associated with oral diseases [94]. Specifically, periodontal disorders have a complex microbiology. Keystone bacterial pathogens including *Porphyromonas gingivalis* and *Aggregatibacter actinomycetem-comitans* physically interact with *Candida species*, according to in vitro investigations [95]. *Bifidobacterium dentium*, *Bifidobacterium adolescentis*, *Streptococcus mutans*, *Scardovia wiggsiae*, *Bifidobacterium longum*, *Selenomounas* spp., *Prevotella* spp., and *Lactobacillus* spp. are the microbes linked to caries, according to the most recent molecular biology techniques [96]. Concerning endodontic infections, in a relatively recent study, Gomes, Fidel, and de Moura Sarquis (2010) isolated filamentous fungi from more than a quarter of root canals of teeth with pulp necrosis and identified *Aspergillus* spp. (*ustus*, *granulosus*, *niger*, and *sydowii*), *Emericella quadriluniata* (a sexual form of *Aspergillus*), *Penicillium species* (*implicatum*, *micsynvisk*, *lividum*, and *citrionigrum*), *Fusarium* (*moniliforme* and *melanochorum*), *Aureobasidium pullulans*, *Exophiala jeanselmei*, *Eurotium amstelodame*, and *Cladosporium sphaerospermum* [95].

Viral infections of the oral cavity, including herpesvirus infections, are common, especially in immunocompromised people [97]. Three of the major oral diseases are halitosis, often caused by poor oral hygiene, the presence of pathogenic bacteria, and free and available iron that promotes bacterial growth; and gingivitis and periodontitis, inflammatory conditions related to the accumulation of bacterial plaque and subsequent gingival inflammation [98]. Lactoferrin treatments can reduce inflammation, restore iron homeostasis, and prevent these most common oral pathological conditions.

Lactoferrin’s properties also make it useful during orthodontic treatments. Its antimicrobial action, which helps reduce the growth of pathogenic bacteria in the mouth, is important in patients with orthodontic appliances because they are more susceptible to oral hygiene problems and caries. Having anti-inflammatory action, it plays an important role in reducing inflammation of the gums and oral mucosa, helping to maintain a healthy oral environment for patients undergoing orthodontic treatment. Last but not least, its wound healing ability may be relevant for patients with oral injuries that have arisen due to the presence of orthodontic appliances in the mouth [99].

In the study by Martin et al., 2016, the antioxidant properties of a gel based on essential oils were studied. Reductions of 21.8% in bleeding on probing (BOP) and 9.0% in the gingival index (GI) were observed in the treatment group compared to the control group. The treatment group also showed improvements in pocket depth (PD), which increased significantly after discontinuation of the gel, confirming the effect of the treatment on gingival health [77]. The level of bacterial plaque was also positively influenced by the treatment with the essential oil gel. The treatment group recorded an 8.4% decrease in the plaque index (PI) while in the control group it increased by 17%. In summary, the gel was shown to be effective in reducing gingival inflammation in orthodontic patients, with improvements in clinical parameters such as BOP, GI, and PI. This treatment could help reduce the risk of bracket loss and white spot lesions associated with gingival inflammation in at-risk orthodontic patients [77,100]. Microbiological research has found a significant increase in the number of bacteria after the application of fixed orthodontic brackets, which leads to imbalances in the oral environment and increases the risk of diseases, especially tooth decay [101]. Generally used chlorhexidine can cause unwanted side effects, but, for Kamath et al., aloe vera could be a natural alternative for plaque control and the treatment of gingivitis without the side effects associated with chlorhexidine [78,102]. Aloe vera is described as a plant with numerous medicinal benefits and its effective use in treating various oral and dental conditions is mentioned [78]. Also, Leiva-Cala et al., 2020 compared the clinical efficacy of aloe vera gel (80%) versus commercial 0.12% chlorhexidine (CHX) gel to prevent traumatic oral ulcers in patients with fixed orthodontic appliances. The results showed that aloe vera gel provided better results than CHX [80]. These studies have highlighted the mechanisms of action of aloe vera which has the following:

- Anti-inflammatory properties thanks to the content of acemannans and anthraquinones which can reduce inflammation by acting on inflammatory processes at a cellular level [103];
- Antioxidant properties thanks to the presence of vitamins C and E and flavonoids which protect cells from damage caused by free radicals [51,104];
- Antimicrobial action preventing infections and helping to maintain oral hygiene [80,105];
- Effects on bacterial plaque, influencing its growth and formation [106];
- Effects on the immune system by helping to regulate the immune response and reduce inflammation [107];
- Positive effects on wound healing by stimulating collagen production especially in the presence of small wounds or irritations caused by orthodontic devices [108,109];
- Hydrating and soothing effects on skin and oral mucous membranes, improving patient comfort and reducing irritation [110,111].

Another comparison with CHX was made in research conducted by Goes in 2016, with Matricaria chamomilla showing antimicrobial and anti-inflammatory properties and not causing gingivitis [112]. Matricaria is a member of the Asteraceae family, a flowering plant widespread in Europe and Asia. Matricaria species include a variety of phytochemicals, including sesquiterpene lactones such as matricin, volatile terpenoids (such as α-bisabolol, bisabolol oxide A and B, β-trans-farnesene, and chamazulene), and phenolic compounds (flavonoids, coumarins, and phenolic compounds) [83]. Studies on the effectiveness of chamomile extracts as mouthwashes revealed that these herbal extracts were beneficial due to their antibacterial and anti-inflammatory qualities. Apigenin is the flavonoid to which anti-inflammatory properties have been entrusted [113]. It is mainly found in natural sources as apigenin-7-glucoside (APG), a glycosylated form. Other primary chemicals implicated in this activity include acid derivatives. The study compared the effects of a mouthwash with Matricaria chamomilla at 1% with a mouthwash with CHX (chlorhexidine) at 0.12% (considered the gold standard), and it emerged that both the VPI (visible plaque index) and the GBI (gingival plaque bleeding index) decreased significantly with Matricaria chamomilla 1%, similar to the effects of CHX 0.12%, in patients with braces-associated gingivitis [83]. Numerous studies have long since proven that natural antioxidants have antibacterial properties. In order to avoid antibiotic resistance, they can work in concert with antibiotic therapy.

Bees are the natural source of propolis. Due to its multiple pharmacological qualities, it has been widely used in folk medicine since ancient times and shows promise as a topical treatment agent. Based on its physico-chemical characteristics (color, consistency, or chemical composition), propolis can be classified among natural products with a medical effect [114]. One type of propolis documented in the literature is Brazilian red propolis (BRP), which includes terpenes, isoflavonoids, pterocarpans, chalcones, flavonoids, prenylated benzophenones, and tannins [49,79]. It possesses antibacterial and anticaries properties, particularly against S. mutans and Lactobacillus species, as well as antifungal, anti-inflammatory, immunomodulatory, antioxidant, and antiproliferative properties thanks to its exceptionally high content of flavonoids and isoflavonoids. Within four weeks, in a 2021 saliva collection test study by Lotif et al., toothpaste containing BRP dramatically reduced the visual plaque index, or VPI, and showed antibacterial action against salivary *Lactobacillus* spp. [79].

A 2014 study by Santamaria evaluated the effectiveness of a tea tree-based dental gel in controlling biofilm and demonstrated positive results, but some patients had difficulty accepting the taste. The use of natural products such as tea tree could be a solution for controlling dental biofilm [115]. The text suggests that the use of these alternatives could revolutionize the treatment and prevention of oral diseases, especially in orthodontic patients [81].

A natural five-carbon sugar alcohol that comes from plants and agricultural products is called xylitol. The term “xylose” (wood sugar), originally used to produce xylitol, is related to the name “xylene”, which comes from the specific structure of the hardwood that produces xylose. Since the early 1960s, it has been included in the diet of diabetic patients, used in infusion treatment for postoperative, burn, and shock patients, and, more recently, it has been used as a sweetener in products intended to promote better dental health [82,116]. Xylitol causes the energy production mechanisms of *Streptococcus mutans* to malfunction, resulting in cell death. Xylitol has a direct inhibitory action, inhibits enamel demineralization (which reduces acid production), and decreases plaque development and bacterial adhesion (i.e., it is antimicrobial) [116]. Masoud’s 2015 study, which considered xylitol as a possible natural product for reducing dental plaque, suggests that xylitol may not offer a significant advantage over standard oral hygiene practices in preventing tooth decay in orthodontic patients, but it still did not have negative effects. A lot of research has been conducted on xylitol in recent years. Clinical studies examining how xylitol affects the development of dental cavities all agree that it is not carcinogenic and that replacing sucrose in chewing gum and sweets with xylitol has positive benefits. Our results demonstrated that chewing gum and chewable tablets containing xylitol do not damage orthodontic appliances or increase the risk of tooth decay. This is in line with another previous research study. Despite the non-cariogenicity of xylitol, we cannot support its use as a cavity prevention strategy because so far it has not had better results than a control group that received regular dental cleanings, oral hygiene education, and check-ups every 6 months [82].

Also, the use of resveratrol (RSV) has become really important in orthodontics, thanks to the possibility of this molecule to promote a reduction in gingival inflammation, through mechanisms with anti-inflammatory, antioxidant, anti-aging, anti-diabetic, anticoagulant, and apoptotic properties [3,117].

In more detail, resveratrol can help reduce inflammatory processes in chronic disorders, protect cells from inflammation, as demonstrated by Diego de Sá Coutinho et al. [118], and it significantly inhibits the release of proinflammatory markers; and with the suppression of NFkB in the large glands, there is a reduction in IL-6 secretion. Such a decrease in IL-6 levels may restrict macrophage STAT3 activity and cause the inflammatory disruption of the inflammatory cascade [119].

In summary, the articles examined show how there are different natural products that can effectively reduce O.S., thanks to their antioxidant, antimicrobial, anti-inflammatory, and wound healing properties.

The qualities of lactoferrin, essential oil gel, aloe vera, Matricaria chamomilla, propolis, tea tree-based gel, xylitol, and resveratrol are examined. These can be of great help in reducing O.S. during orthodontic treatment.

## 5. Conclusions

O.S., characterized by an imbalance between oxidizing and antioxidant agents, can harm tissues and cells. Orthodontic alloys release metal ions, with nickel-titanium archwires releasing more ions than stainless steel, affecting the oral oxidative–antioxidant balance temporarily. The study found that fixed orthodontic treatment induces short-term systemic O.S. but generally returns to normal after 24 weeks. Further research is needed to understand the impact of orthodontic interventions on saliva composition. Additionally, natural products like aloe vera, tea tree, and Matricaria chamomilla have potential in reducing gingival inflammation in orthodontic patients. Aloe vera controls plaque effectively, tea tree controls biofilms, and Matricaria chamomilla has antimicrobial and anti-inflammatory properties, offering promise for oral disease treatment and prevention in orthodontic patients. Other natural products are important in maintaining low oral pathogenic bacterial load, such as lactoferrin or in reducing inflammation, even during orthodontic treatment, such as phenols and resveratrol. Further studies are needed to evaluate the effectiveness and actual action of these products in order to reduce oxidative stress as much as possible during orthodontic treatment. 

## Figures and Tables

**Table 1:** Database search indicators.

Article screening Strategy	Database: Scopus, Web of Science, and Pubmed
	Keywords: A “Natural Products”; B “Oxidative Stress”; C “Orthodontic”
	Boolean variable: “AND” and “OR”
	Timespan: 2013–2023
	Language: English

**Table 2:** Characteristics of the studies included in the analysis.

Authors (Year)	Study Design	Materials and Methods	Outcomes
Mario Portelli et al., 2017 [69]	Experimental	Twenty-three 12- to 16-year-old patients receiving multibracket self-ligating vestibular orthodontic appliances participated in the study. Salivary specimens collected at T1, T2, and T3.	- Repeated measures showed no significant time point differences. - No correlation found between dental hygiene and O.S. Orthodontic treatment with multibracket vestibular metallic appliances did not affect O.S. during the first ten weeks.
Vito Kovac et al., 2020 [70]	Experimental study	Compositions of stainless steel, nickel-titanium, cobalt-chromium, and β-titanium orthodontic alloys were simulated with mixtures of Fe, Ni, Cr, Co, Ti, and Mo metal ions to assess cytotoxicity and O.S. in Saccharomyces cerevisiae yeast strains.	- Cobalt-chromium alloy cytotoxic at 100 µM. - Reactive oxygen species and oxidative damage detected in stainless steel and both cobalt-chromium alloys at 1000 µM (wild-type yeast) and 100 µM (ΔSod1 and ΔCtt1 mutants). - In no yeast strain did simulated nickel-titanium or β-titanium alloy cause O.S.
Vito Kovac et al., 2022 [71]	Experimental	Orthodontic materials (archwires, brackets, and molar bands) were incubated in artificial saliva for 90 days. Metal composition and ion release were assessed. Metal ion mixtures were prepared to assess O.S., antioxidant enzyme defense, and protein damage.	- Beta titanium alloy released the fewest metal ions without inducing O.S. or protein damage. - Only at high concentrations could O.S. and protein damage result from stainless steel and cobalt-chromium alloy. - All orthodontic alloys affected antioxidant enzyme activity, but concentrations were below the maximum tolerated dose for oxidative stress.
Piotr Buczko et al., 2017 [72]	Experimental study	Included 37 volunteers, saliva samples collected immediately before, one week after, and twenty-four weeks after appliance insertion.	- Increased nickel concentration in saliva one week after treatment.
Lucía Angeles-Estrada et al., 2023 [73]	Experimental study	Oral epithelial cells from 51 healthy volunteers receiving orthodontic treatment. Sampling: Samples taken before treatment, at 6 months, and at 9 months. OS Assessment: Quantification of 8-hydroxy-2’deoxyguanosine (8-OHdG) levels. Gene Expression: Evaluation of antioxidant enzymes superoxide dismutase (SOD) and catalase (CAT). Genotoxicity Assessment: Multiplex polymerase chain reaction (PCR) and fragment analysis for DNA degradation and instability.	8-OHdG increased during treatment, but the increase was not statistically significant. SOD increased by 2.5-fold at 6 months and 2.6-fold at 9 months. CAT increased by threefold at 6 months and returned to baseline levels at 9 months. DNA degradation found in 8% and 12% of DNA samples at 6 and 9 months, respectively. DNA instability detected in 2% and 8% of DNA samples at 6 and 9 months, respectively.
Cristina Menéndez López-Mateos et al., 2022 [74]	Randomized clinical trial.	Participants: Non-randomized clinical trial with consecutively recruited patients over 18 years of age undergoing orthodontic treatment. Groups: Group A (48 patients) treated with clear aligners (Invisalign^®^), and Group B (19 patients) treated with Damon System^®^ 0.22″ self-ligating brackets with light forces. Saliva Samples: collected at the beginning of treatment, 30 days later, and 90 days later by a single physician Analyzed Parameters: AOPP, TAC, and MPO levels.	AOPPs levels increased in both groups after the initial 30 days of orthodontic treatment with clear aligners or fixed self-ligating brackets. During the first stages of orthodontic therapy, whether using fixed brackets or clear aligners, there were no appreciable changes in TAC and MPO levels. During the first ninety days of therapy, there were no discernible changes in AOPP levels between self-ligating brackets and transparent aligners. Salivary antioxidant capacity remained similar between the two orthodontic techniques during the initial treatment phases.
Sevil Sema Atuğ Özcan et al., 2014 [75]	Experimental Study	Fifty volunteers who needed orthodontic treatment with fixed appliances participated in the trial. Saliva and GCF samples were taken prior to therapy, one month into the regimen, and six months into it. Periodontal clinical parameters were measured, and biochemical parameters (IL-1 β, TNF-α, 8-OHdG, NO, and MDA) were analyzed using ELISA and spectrophotometric methods.	IL-1 β level in GCF at the 6th month was significantly different from the baseline (*p* < 0.05). However, all other biochemical parameters in both saliva and GCF did not exhibit significant changes at any measurement period.
Vito Kovac et al., 2019 [17]	Experimental Comparative Study	A total of 54 male participants with malocclusion were randomly assigned to either the treatment group (TG; *n* = 27) or control group (CG; *n* = 27). Capillary blood was collected at various time points.	Short-term systemic O.S. was brought on by orthodontic treatment with fixed appliances; this stress resolved seven days following the placement of the archwire.
Cigdem Guler et al., 2015 [76]	Experimental study	- Thirty children divided into three groups. - Orthodontic composites: Transbond XT, Kurasper F, and GrenGloo. - Salivary TOS, TAS, and 8-OHdG levels measured at three time points: T1 (before treatment), T2 (1 month after appliance placement), and T3 (3 months after appliance placement).	- There are no appreciable variations in 8-OHdG, TOS, or TAS between the three orthodontic composites. - TAS decreased over time in all composite groups. Significant decreases observed for Kurasper F and GrenGloo. - Salivary cytotoxicity markers were not elevated by fixed orthodontic equipment using evaluated composite materials.
Martin et al., 2016 [77]	Randomized controlled trial	Thirty orthodontic patients were screened for gingivitis and divided into treatment and placebo groups. They underwent periodontal examinations and were instructed to apply a topical gel twice daily, then discontinue use.	Patients with gum disease can successfully reduce inflammation by using an anti-inflammatory gel made of essential oils to their teeth.
Kamath et al., 2023 [78]	Randomized controlled trial	A total of 30 participants with fixed orthodontic treatment, divided into test and control groups. Results were analyzed using Student’s paired and unpaired *t*-tests, focusing on plaque index, gingival index, and bleeding on probing.	Larger multicentric trials are required; however, aloe vera, the gold standard mouthwash, showed promising effectiveness in lowering plaque and gingivitis scores without harmful consequences.
Lotif et al., 2022 [79]	Double-blind randomized clinical trial	A total of 42 participants randomized into two groups based on dentifrice, recorded saliva, and microbiological analysis identified *Lactobacillus* spp. isolates, with VPI and CFU/mL values.	The toothpaste containing BRP demonstrated antimicrobial properties against Lactobacillus spp. and reduced the VPI for up to four weeks.
Leiva-Cala et al., 2020 [80]	Randomized clinical trial	A study involving 140 patients aged 12 and older with permanent teeth found that 43.6% of transactions were TOUs. In the CHX arm, 81.4% of patients experienced TOUs, while 5.7% of patients treated with aloe vera gel did not suffer from TOUs.	Administration of aloe vera gel may be essential in shielding patients with permanent orthodontic equipment from traumatic mouth ulcers.
Santamaria et al., 2014 [81]	Comparative Study	A study of 34 volunteers, divided into two groups, monitored their dental hygiene for four weeks. They were given different treatments for plaque index and bacteria count, and after 15 days, they were instructed to return to their usual habits. The data was analyzed statistically with a 5% significance level.	The use of natural products such as melaleuca could be a solution for controlling dental biofilm, especially in orthodontic patients.
Masoud et al., 2015 [82]	Comparative Study	Considered xylitol as a possible natural product for reducing dental plaque, suggests xylitol may not offer significant benefit.	Xylitol may not offer a significant benefit, but it still had no adverse effects.
Goes et al., (2016) [83]	Pilot Study	The study compared the effects of mouthwash with 1% Matricaria chamomilla, with a mouthwash with 0.12% CHX (chlorhexidine) (considered the gold standard).	It was found that both VPI (visible plaque index) and GBI (gingival plaque index) bleeding indexes significantly decreased with MTC at 1%, similar to the effects of CHX at 0.12%, in patients with gingivitis associated with orthodontic appliances.

## Data Availability

Not applicable.

## Abbreviations

AD	antioxidative defense
AOPPs	advanced oxidative protein products
BOP	bleeding on probing
BRP	Brazilian red propolis
CAT	catalase
CG	control group
CHX	chlorhexidine
FPN	ferroportin
GBI	gingival plaque bleeding index
GCF	gingival crevicular fluid
GI	gingival index
MPO	myeloperoxidase activity
MTC	matricaria therapy chamomilla
NI-TI	nickel-titanium
NO	nitric oxide
OS	oxidative stress
PCR	polymerase chain reaction
PD	probing depth
PI	plaque index
ROS	reactive oxygen species
SOD	superoxide dismutase
TAC	total antioxidant capacity
TBARS	thiobarbituric acid reactive substances
TG	treatment group
TOS	total oxidant status
VPI	visible plaque index
